# A three-component Breakfast Quality Score (BQS) to evaluate the nutrient density of breakfast meals

**DOI:** 10.3389/fnut.2023.1213065

**Published:** 2023-09-28

**Authors:** Romane Poinsot, Matthieu Maillot, Gabriel Masset, Adam Drewnowski

**Affiliations:** ^1^MS-Nutrition, Marseille, France; ^2^Cereal Partners Worldwide, Lausanne, Switzerland; ^3^Center for Public Health Nutrition, University of Washington, Seattle, WA, United States

**Keywords:** breakfast, nutrients, score, nutritional recommendations, meal, nutrient density, nutrient profiling

## Abstract

**Background:**

Nutrient profiling methods can be applied to individual foods or to composite meals. This article introduces a new method to assess the nutrient density of breakfast meals.

**Objective:**

This study aimed to develop a new breakfast quality score (BQS), based on the nutrient standards previously published by the International Breakfast Research Initiative (IBRI) consortium.

**Methods:**

BQS was composed of three sub-scores derived from the weighted arithmetic mean of corresponding nutrient adequacy: an eLIMf sub-score (energy, saturated fat, free sugars, and sodium), a PF (protein and fiber) sub-score, and a VMn_1 − 14_ micronutrient sub-score, where *n* varied from 0 to 14. The effects of assigning different weights to the eLIMf, PF, and VMn were explored in four alternative models. The micronutrients were calcium, iron, potassium, magnesium, zinc, vitamin A, thiamin, riboflavin, niacin, vitamin B5, vitamin B6, vitamin B12, vitamin C, and vitamin D. Micronutrient permutations were used to develop alternate VMn_1 − 14_ sub-scores. The breakfast database used in this study came from all breakfasts declared as consumed by adults (>18 years old) in the French dietary survey INCA3. All models were tested with respect to the Nutrient Rich Food Index (NRF9.3). BQS sensitivity was tested using three prototype French breakfasts, for which improvements were made.

**Results:**

The correlations of the models with NRF9.3 improved when the VMn_>3_ sub-score (n > 3) was included alongside the PF and eLIMf sub-scores. The model with (PF+VMn) and eLIMf each accounting for 50% of the total score showed the highest correlations with NRF9.3 and was the preferred final score (i.e., BQS). BQS was sensitive to the changing quality of three prototype breakfasts defined as tartine, sandwich, and cereal.

**Conclusion:**

The proposed BQS was shown to valuably rank the nutritional density of breakfast meals against a set of nutrient recommendations. It includes nutrients to limit along with protein, fiber, and a variable number of micronutrients to encourage. The flexible VMn sub-score allows for the evaluation of breakfast quality even when nutrient composition data are limited.

## 1. Introduction

The nutritional value of the breakfast meal can be assessed in a number of ways (1, 2). In their simplest form, scores of breakfast quality award points to food groups or nutrients to encourage and subtract points for food groups and nutrients to limit. A 10-point breakfast quality index (BQI) published in 2012 (2) awarded points for breakfast energy in the range of 20–25% of total daily intake and for the presence of cereals, fruits/vegetables, dairy products, monounsaturated fats, and calcium. An additional point was given for having cereals, fruit/vegetables, and dairy in the same meal (2). The same BQI subtracted points for the breakfast content of sugars and saturated and trans fats (2).

Nutrient profiling (NP) methods generally use both positive and negative components to assess the nutrient density of meals (3), dishes (4), or individual foods (5). The Nutri-Score (6), the Health Star Rating (7), and the Nutrient Rich Food Index (NRF9.3) (7) each have positive and negative sub-scores. Negative sub-scores are typically driven by saturated and trans fats, total or added sugars, and sodium, but can also include energy (6). While the choice of nutrients to encourage can vary, the Nutri-Score, Health Star Rating, and NRF9.3 each include both protein and fiber (6–8). The Nutri-Score does not include any vitamins or minerals, preferring to award points based on the foods' content of pre-selected food groups (fruit, vegetables, and nuts) (6). By contrast, the NRF9.3 score complements protein and fiber with calcium, iron, potassium, magnesium, vitamin A, vitamin C, and vitamin E (now replaced by vitamin D) (7). Depending on the NP model, the number *n* of vitamins and minerals has varied from 3 to as many as 23 (9, 10).

The International Breakfast Research Initiative (IBRI) has proposed a set of nutrient standards to assess the nutritional value of breakfast meals (11, 12). In a series of IBRI studies, the breakfast quality of representative populations in the United States (13), Canada (14), France (15), Spain (16), the United Kingdom (17), and Denmark (18) was measured using the Nutrient Rich Food Index (7). Other studies have since explored the nutritional contributions of the breakfast meal in Latin America (19, 20) and the Philippines (21). The IBRI targets for vitamins and minerals at breakfast were based on actual consumption levels during breakfast and on overall nutrient adequacy (22).

The present goal was to simplify the large set of nutrient-by-nutrient IBRI recommendations into an overall breakfast quality score (BQS). Alternative scores were all comprised of the same three components. The negative sub-score was eLIMf (energy, saturated fat, free sugars, and sodium). The two positive sub-scores were PF (protein and fiber) and VMn, the latter composed of a variable number of vitamins and minerals. As in other NP models, the final quality score was based on the difference between the negative and positive sub-scores (6–8). The present innovation was to vary the number of vitamins and minerals in VMn from 0 to 14 and to explore the impact of micronutrient permutations (9, 10). We also explored the effects of assigning differential weights to the eLIMf, PF, and VMn sub-scores on the total score. This was done because the weighting of the sub-scores affects the final evaluation (23). Although some NP models are driven by negative elements, energy, sugar, and fat, others are weighted to favor protein, fiber, vitamins, and minerals.

Using the French National Dietary Survey INCA3, alternative scores were tested with respect to the NRF9.3 nutrient density score. The nutrient density of the breakfast meal was then compared across the tertiles of BQS. BQS sensitivity to changes in breakfast composition was tested using three French breakfasts, identified as tartine, sandwich, and cereal.

## 2. Methods

### 2.1. INCA 3 and CIQUAL databases

Dietary data on breakfast consumption in France came from the INCA3 study on dietary intakes of the French population (24). INCA3 is based on three non-consecutive 24 h dietary recalls for 1,907 men and 2,207 women (2 weekdays and 1 weekend day). Only data for adults aged ≥18 years (*n* = 2,121) were analyzed. As part of the dietary intake assessment, participants needed to name the eating occasion. There were 10 possibilities (including “breakfast”). Only foods consumed during the declared breakfast meal were analyzed. Breakfasts consisting of coffee or tea only (sweetened or not) were excluded. A total of 4,478 breakfasts were analyzed.

The associated ANSES CIQUAL 2016 nutrient composition database (25) provides energy and nutrient values per 100 g edible portion for all the foods consumed by INCA3 participants. Free sugars were estimated from added sugars (26). Portion sizes were based on a previous study in France (27).

### 2.2. Development of the breakfast quality score

#### 2.2.1. Establishing nutrient standards for BQS

Table 1 summarizes the nutrient standards developed by IBRI (11, 12). Also shown are the formulas for calculating nutrient adequacy. Upper and lower bounds for energy were set for the breakfast meal following IBRI recommendations. Maximum recommended values (MaxRV) were set for saturated fats, free sugars, and sodium. Minimum recommended values (MinRV) were set for protein, fiber, and 14 micronutrients.

**Table 1:** Calculation of nutrient adequacy for breakfast quality score.

**Sub-score**	**Nutrient**	**Unit**	**Reference values**		**Boundaries**		**Percentage of adequacy**
			**MinRV** ^*^	**MaxRV** ^*^	**Lower bound**	**Upper bound**	
eLIMf	Energy	kcal	300	500	0	500 + 300 = 800	$Adeq_{i}=\min\left(\frac{obs_{i}\;}{rec\overset{\min}{\underset{i}{o}}}\times 100,\frac{upper_{i}-obs_{i}\;}{upper_{i}-reco_{i}^{\max}}\times 100,\;100\right)$
	SFAs	%EBI		10		2 × 10 = 20	
	Free sugars	%EBI		10		2 × 10 = 20	
	Sodium	mg		400		2 × 400 = 800	
PF	Proteins	g	10		0		$Adeq_{i}=\min\left(\frac{upper_{i}-obs_{i}\;}{upper_{i}-reco_{i}^{\max}}\times 100,\;100\right)$
	Fibers	g	5		0		
VMn	Calcium	mg	250		0		$Adeq_{i}=\min\left(\frac{obs_{i}\;}{rec\overset{\min}{\underset{i}{o}}}\times 100,\;100\right)$
	Iron	mg	2.8		0		
	Magnesium	mg	62		0		
	Potassium	mg	700		0		
	Zinc	mg	2.2		0		
	Vitamin A	mg	80		0		
	Vitamin B1	mg	0.24		0		
	Vitamin B2	mg	0.36		0		
	Vitamin B3	mg	3.75		0		
	Vitamin B6	mg	0.26		0		
	Folate	μg	80		0		
	Vitamin B12	μg	0.48		0		
	Vitamin C	mg	20		0		
	Vitamin D	μg	1		0		

*obs*_*i*_, observed breakfast intake of nutrient *i*; $reco_{i}^{\min}$, minimum recommended value for nutrient *i*; *upper*_*i*_, upper bound for nutrient *i*; $reco_{i}^{\max}$, maximum recommended value for nutrient *i*; EBI, energy breakfast intake; n, number of micronutrients. Free sugars refer to all monosaccharides and disaccharides added to foods by the manufacturer, cook, or consumer, and sugars naturally present in honey, syrups, and fruit juice. ^*^The MinRV and MaxRV values came from IBRI recommendations for adults and were based on 10 or 20% of daily recommendations from WHO/Codex.

IBRI-derived MinRV and MaxRV values for adults were based on 10 or 20% of daily recommendations from WHO/Codex (28) and from the WHO guidelines for nutrients of public health concern (free sugars, saturated fats, and sodium) (29). For breakfast energy, a maximum adequacy score of 100% was achieved when breakfast energy intakes fell within the 300–500 kcal range. The lower bound (0%) was set at 0 kcal. The upper bound (also 0%) was set at 800 kcal, i.e., 500 kcal + 300 kcal. When breakfast energy was lower than 300 kcal or higher than 500, the adequacy scores diminished in proportion to intakes relative to the recommended values. When the breakfast energy was higher than the upper bound (800 kcal), the adequacy score became negative.

For saturated fat, sodium, and free sugars, a maximum adequacy score (100%) was achieved when breakfast intakes were <MaxRV. The upper bound (0%) was set at 2 × MaxRV. Adequacy diminished in proportion from MaxRV to the upper bound (2 × MaxRV). When the observed values at breakfast were higher than the upper bounds, the adequacy scores became negative.

For protein, fiber, vitamins, and minerals, a maximum adequacy score (100%) was achieved when breakfast nutrient intakes were >MinRV. The lower bound (0%) was set at zero consumption. Nutrient adequacy between these two points was calculated by dividing the breakfast value by the recommended value and multiplying by 100.

#### 2.2.2. Definitions of BQS sub-scores

Nutrient adequacies were grouped into one of three sub-scores: eLIMf, PF, or VMn (Table 1). The sub-scores were equal to the arithmetic mean of corresponding nutrient adequacies (Equations 1–3). The eLIMf sub-score (Equation 1) included energy, saturated fat, sodium, and free sugars (rather than total or added) adequacies. The eLIMf sub-score could be either negative or positive. The lower the content of nutrients to be limited, the higher the eLIMf sub-score.


(1) $$\begin{array}{l} \;eLIMf=\frac{1}{4}\times \;\sum_{i=1}^{i=4}Adeq_{i},\\ i=\left\{energy,\;saturated\;fats,\;free\;sugars,\;sodium\right\},\\ \;eLIMf\;\in \;[-\infty ;100] \end{array}$$

The second sub-score was named PF (Equation 2) since it contained protein and fiber adequacy.


(2) $$\begin{array}{l} \;PF=\;\frac{1}{2}\times \sum_{i=1}^{i=2}Adeq_{i},\\ i=\left\{proteins,\;fibers\right\},\;PF\;\in [0;100], \end{array}$$

In previous studies, NP profiles have used a wide range of micronutrients (9, 10). The third sub-score VMn (Equation 3) was based on a variable number n of vitamins and minerals (n ranges from 0 to 14) adequacies. The 14 micronutrients for which IBRI recommendations were available were calcium, iron, potassium, magnesium, zinc, vitamin A, thiamin, riboflavin, niacin, vitamin B5, vitamin B6, vitamin B12, vitamin C, and vitamin D. The VMn sub-score could only be positive.


(3) $$\begin{array}{l} \;VMn=\frac{1}{n}\sum_{i=1}^{i=n}Adeq_{i},\\ i\;\in \left\{0,\;micronutrient_{1},\ldots \;micronutrient_{n}\right\},\\ \;n\;\leq 14,\;VMn\;\in \left[0;100\right] \end{array}$$

All possible combinations of the 14 micronutrients were systematically tested. The number of possible combinations of micronutrients depended on their number. For VM = 0 and for VM = 14, there was only one possible option. For VM = 1 and for VM = 13, there were 14 possible choices. For VM = 2 and for VM = 12, there were 91 possible combinations of micronutrients. For VM = 3 and VM = 11, there were 364 and so on. For VM = 7, there were 3,432 possible combinations.

#### 2.2.3. Alternative BQS models with different sub-score weights

Alternative BQS models were calculated as a weighted mean of the three sub-scores following Equation 4. In every case, the minimum score was 0, so there were no negative scores. Scores ranged from 0 to 100, with the highest scores given to those breakfasts that met all of the IBRI recommendations.


(4) $$\begin{array}{l} Breakfast\;quality\;score=\;\alpha \times \;eLIMf+\;\beta \;\times PF+\gamma \times VMn\; \end{array}$$

Where α, β, and γ are weights ranging from zero to 1, and their sum is equal to 1.

Four alternative BQS models were tested, each with a different sub-score weighting scheme (Table 2).

**Table 2:** Weighting scheme of component sub-scores for the four alternative BQS models (with *n*, the number of micronutrients).

**Alternative models**	**Weight of sub-score**			**Weight of each sub-score, where n ** = 14****
	**eLIMf** (α)	**PM** (β)	**VMn** (γ)	
Balanced model	$\frac{1}{2}$	$\frac{\left(\frac{2}{2+n}\right)}{2}$	$\frac{\left(\frac{n}{2+n}\right)}{2}$	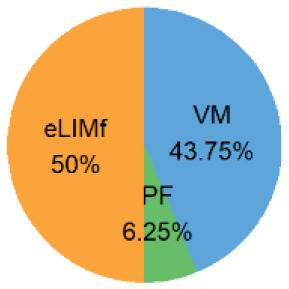
Unweighted model	$\frac{4}{6+n}$	$\frac{2}{6+n}$	$\frac{n}{6+n}$	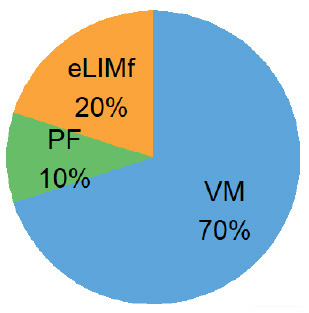
Micronutrient model	$\frac{1}{2}\times \frac{4}{6}=\frac{1}{3}$	$\frac{1}{2}\times \frac{2}{6}=\frac{1}{3}$	$\frac{1}{2}$	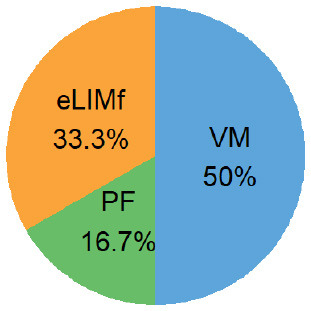
Three-way model	$\frac{1}{3}$	$\frac{1}{3}$	$\frac{1}{3}$	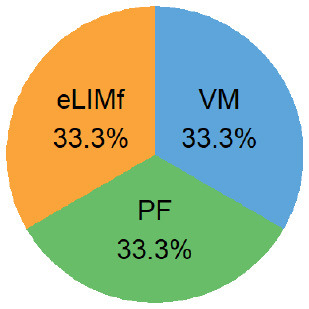

In the balanced model, the eLIMf sub-score accounts for 50% of the BQS, and the sum of PF and VMn sub-scores (PF+VMn), where *n* ≤ 14, also accounts for 50% of the BQS. The weights α, β, and γ differ between models and are shown in Table 2. In the unweighted model, all 6+n elements are equivalent. Each element accounts for [1/(6+n)] × 100%] of the total score as VMn rises from 0 to 14.

In the micronutrient model, the VMn sub-score (*n* ≤ 14) now accounts for 50% of total BQS. The eLIMf and PF sub-scores together account for 50% of the BQS total. In the three-way model, the eLIMf sub-score, the PF sub-score, and the VMn sub-score (*n* ≤ 14) each account for 1:3 of the BQS total.

**Figure 1:**
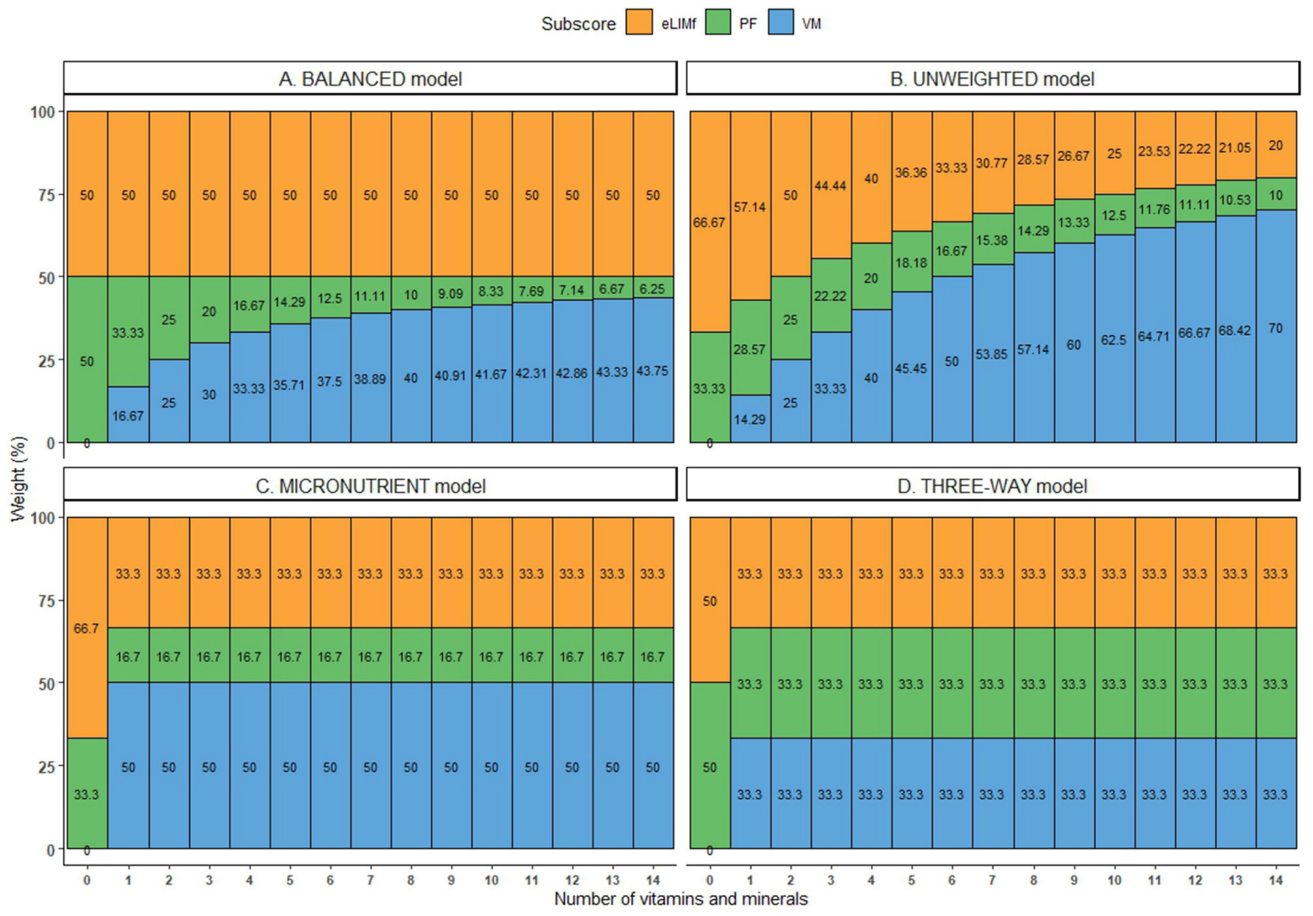
Weight (in %) of each sub-score in the four alternative BQS models, where the number of micronutrients ranged from 0 to 14.

Pie charts are an alternative way of visualizing BQS weights when *n* = 14, and they are presented in the last column of Table 2. Figure 1 shows the shift in weights for the four alternative weighting schemes for BQS sub-scores as the number of vitamins and minerals in the VMn sub-score rises from 0 to 14.

### 2.3. Testing alternative BQS models

Scores obtained using alternative BQS models on the INCA3 breakfast meals were compared to scores for the same breakfast meals generated by the NRF9.3 nutrient profiling model (7) and to scores obtained with the same BQS model where *n* = 14 (called “complete” BQS). The NRF9.3 is the sum of percent daily values (DV) for nine nutrients to encourage (proteins, fibers, calcium, iron, magnesium, potassium, vitamin C, vitamin A, and vitamin D) minus the sum of percent DV for three nutrients to limit (saturated fats, added sugars, and sodium). All values are calculated per 100 kcal and capped at 100% (7). Correlations were estimated using the Spearman rank-order correlation coefficient. For partial VMn scores where *n* < 14, correlations were obtained for each combination of micronutrients, and their distribution was analyzed using boxplots.

The model that correlated best with NRF9.3 was selected and called “BQS” in the rest of the article.

BQS values for 4,478 breakfasts in the INCA3 database were split into tertiles. The general linear model then compared values of energy and nutrients (for which IBRI recommendations were available), sub-scores eLIMf, PF, and VM, and grams of eight dietary components across the BQS tertiles. The eight dietary components of interest were fruits and vegetables, whole-grain, refined-grain, milk and dairy, plant fats, animal fats, sugary foods, and sweet-tasting beverages (e.g., soda and fruit juices). A *post-hoc* comparison (Tukey's HSD test) was performed when the difference was significant.

Statistical analyses used the R software version 4.1. The level of significance was set to 5% for all tests.

## 3. Results

### 3.1. Alternative BQS models with complete VMn sub-scores

Breakfast meals in the INCA 3 database were evaluated using the four alternative BQS models. All four BQS models were highly correlated with each other (range r = 0.76 to r = 0.99; results not shown). With all the alternative BQS models, none of the 4,478 breakfasts got a 100% adequacy score. Mean BQS values ranged from 51.6% (balanced model) to 58.5% (unweighted model) and were comparable for the four models.

**Figure 2:**
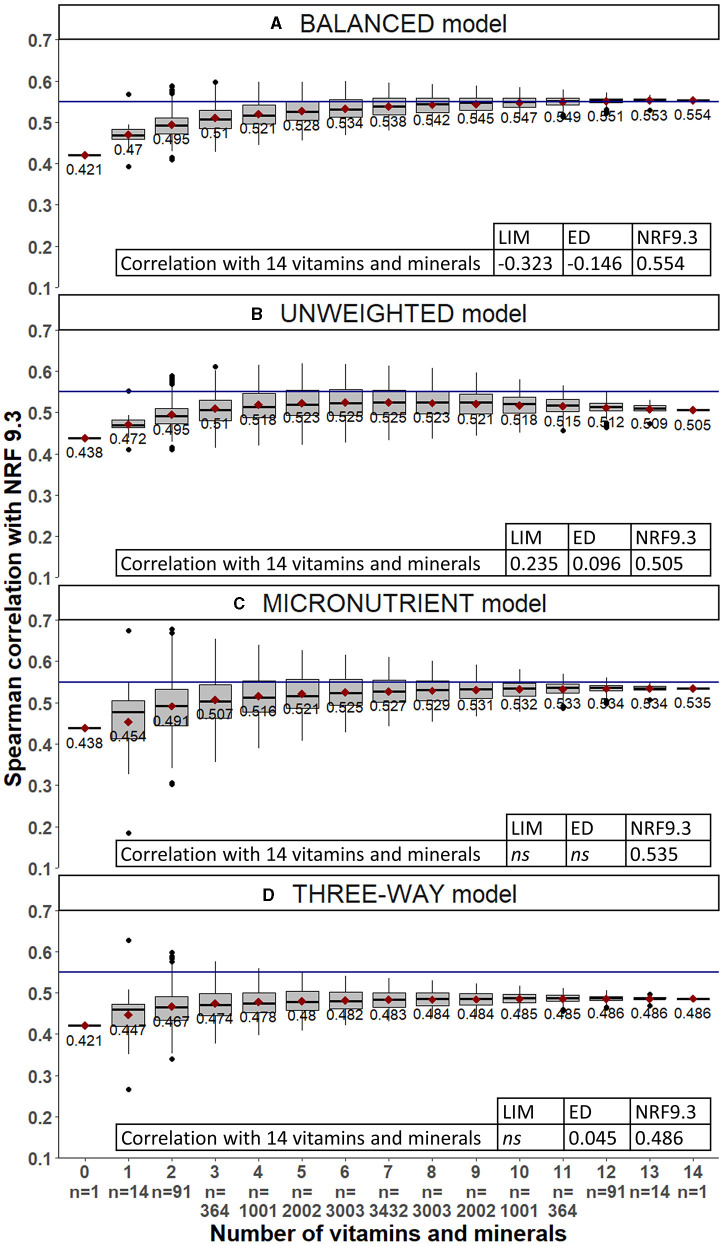
Spearman correlations between each alternative BQS model and NRF9.3 for all combinations of VMn ranging from 0 to 14 micronutrients. The horizontal reference line is set to 0.55.

The balanced model showed the highest correlations with NRF9.3 nutrient density scores (r = 0.55) and the lowest correlations with energy density (r = −0.15) of breakfasts in the INCA database (Figure 2). The balanced model had a moderate correlation with LIM.

### 3.2. Alternative BQS models with partial VMn sub-scores

Figure 2 shows that correlations with NRF9.3 improved as the VMn sub-score incorporated more vitamins and minerals. For all four alternative BQS models, correlations with NRF9.3 were weakest when no micronutrients were included (VM_n = 0_). Correlations improved when the VMn component included at least three vitamins or minerals. For balanced, micronutrient, and three-way models, correlations with NRF9.3 increased when the VMn number of micronutrients increased. For the unweighted model, the correlation with NRF9.3 increased up to 5–6 micronutrients and then decreased. The average correlations of NRF9.3 with the balanced model were very close to those with the micronutrient model; however, according to the permutation, the correlations with the micronutrient model were less homogeneous than those with the balanced model (see the size of boxes in Figure 2).

**Figure 3:**
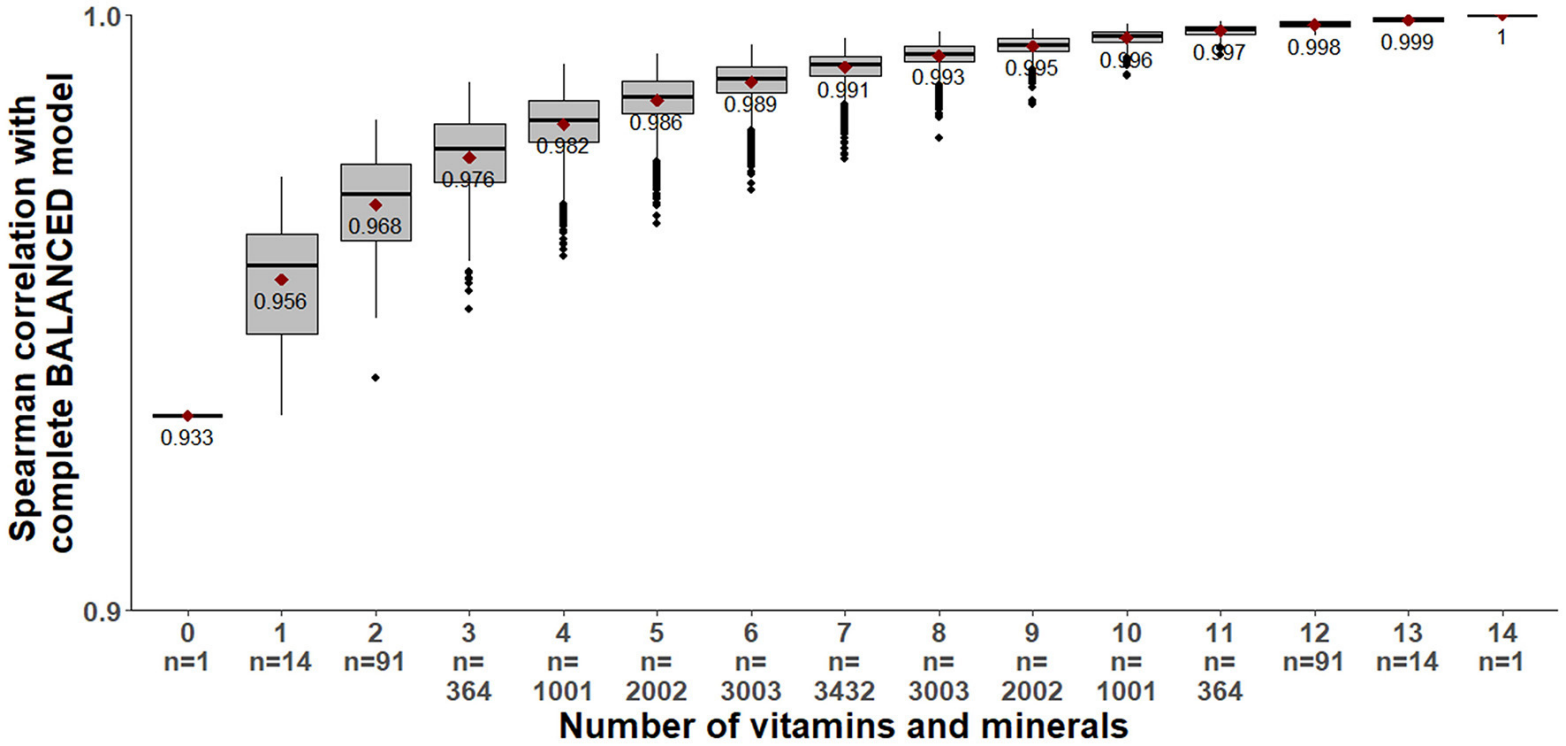
Distribution of Spearman correlation between the complete balanced model (based on 14 micronutrients) and the balanced model when VM ranged from 0 to 14 micronutrients.

All average correlations were highest for the balanced model. For the balanced model, when VM_n = 3_, correlation values ranged from 0.43 to almost 0.60. This variability was related not only to the number but also to specific combinations of micronutrients. According to Figure 3, the correlation between complete and partially balanced BQS stayed high (minimum 0.93 without micronutrients) even when the number of micronutrients included in the score decreased. However, it was not clear which vitamins and minerals were the most important. Based on these results, the balanced model was selected as BQS and was subject to further testing.

### 3.3. Testing the performance of BQS (i.e., the balanced model)

#### 3.3.1. Distribution of BQS in INCA3 adult breakfasts

**Figure 4:**
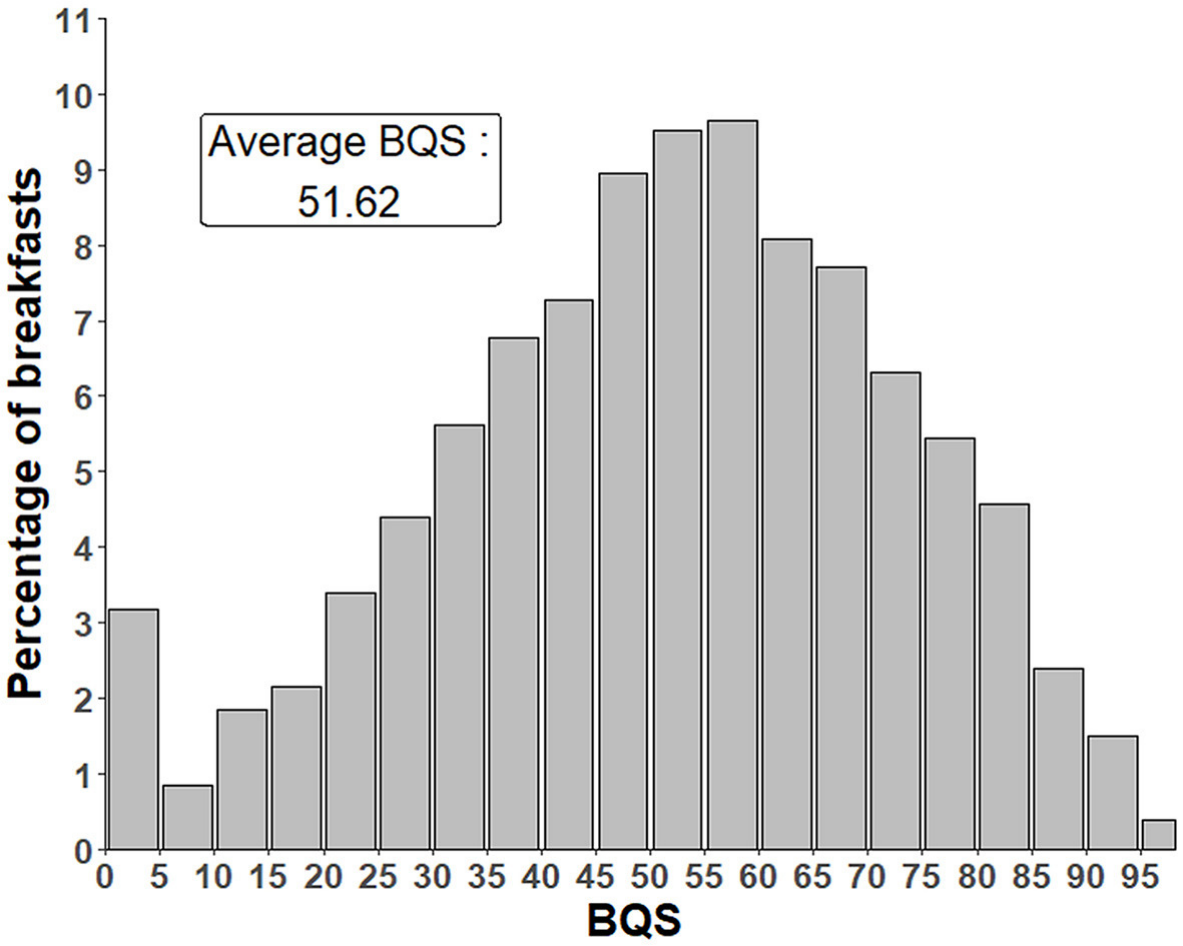
Distribution of BQS in INCA3 adults breakfast. BQS, BQS balanced model.

The distribution of the BQS values in INCA3 adult breakfasts is shown in Figure 4. Only 3% of the breakfasts were cut at 0%. The score of 0% means that the breakfast provided more negative points (from the eLIMf sub-score) than positive points from PF and VM. The BQS distribution exhibits a normal shape. The average (51.6%) and median (53.1%) balanced BQS were close to 50%.

#### 3.3.2. Nutritional and dietary components by tertiles of BQS

Percent nutrient adequacy in the first tertile of BQS ranged from 0 to 43.7% and in the third tertile from 62.1 to 98.4%. Table 3 confirmed that sub-score eLIMf increased as well as sub-scores PF and VM when BQS values increased. Indeed, the amounts of nutrients to limit decreased between low and medium and between medium and high tertiles of BQS, and, except for vitamin A, the amounts of nutrients to encourage increased between low and high tertiles of BQS. For proteins, fiber, iron, magnesium, potassium, and vitamins B1, B3, B9, and C, the difference between the low and medium tertiles was not significant, but the difference between the medium and high tertiles was significant. Likewise, amounts of healthier food groups such as fruits and vegetables, whole-grain foods, milk and dairy products, and plant-based fats were higher. Conversely, amounts of less healthy food groups such as refined-grained foods, animal fats, sugary foods, and sweet-tasting beverages were lower in breakfast in low tertiles than in breakfast in medium or high tertiles. Breakfasts in the highest tertile of BQS were thus of higher nutritional quality than breakfasts in the medium or low tertile of BQS.

**Table 3:** Average BQS^$^, sub-scores, energy, and nutrients included in IBRI recommendations and dietary components by tertiles of BQS.

	**Tertile of BQS**			
	**Low [0; 43.7]**	**Medium [43.7; 62.1]**	**High [62.1; 98.4]**	***p*** **-value**
BQS^$^	27.6	52.9	74.3	^***^
Sub-score eLIMf	−0.18	23.1	35.8	^***^
Energy (kcal)	482^a^	410^b^	411^b^	^***^
SFA (% EBI)	14.6	13.6	11.5	^***^
Free sugars (% EBI)	28.4	16.6	10.6	^***^
Sodium	562	485	438	^***^
Sub-score PF	3.61	4.11	4.98	^***^
Proteins (g)	9.90^a^	10.0^a^	14.3^b^	^***^
Fibers (g)	3.47^a^	3.35^a^	4.19^b^	^***^
Sub-score VM	23.0	25.7	33.5	^***^
Calcium (mg)	130	163	324	^***^
Iron (mg)	1.60^a^	1.65^a^	2.15^b^	^***^
Magnesium (mg)	70.2^a^	73.6^a^	92.7^b^	^***^
Potassium (mg)	597^a^	630^a^	829^b^	^***^
Zinc (mg)	0.95	1.04	1.65	^***^
Vitamin A (μg RAE)	133	92.7	98.2	0.174
Vitamin B1 (mg)	0.25^a^	0.26^a^	0.38^b^	^***^
Vitamin B2 (mg)	0.34	0.41	0.69	^***^
Vitamin B3 (mg)	2.88^a^	3.11^a^	3.68^b^	^***^
Vitamin B6 (mg)	0.21	0.23	0.36	^***^
Vitamin B9 (mg)	56.7^a^	58.8^a^	77.6^b^	^***^
Vitamin B12 (μg)	0.41	0.53	1.09	^***^
Vitamin C (mg)	18.8^a^	18.2^a^	26.4^b^	^***^
Vitamin D (μg)	0.41	0.55	0.97	^***^
**Dietary components**			
Fruits and vegetables (g)	8.38	14.2	34.7	^***^
Whole-grain foods (g)	6.70	9.45	14.3	^***^
Refined-grain food (g)	50.2	38.1	25.2	^***^
Milk and dairy products (g)	47.0	71.9	196	^***^
Plant fats (g)	1.15^a^	2.33^b^	2.80^b^	^***^
Animal fats (g)	7.25	4.92	2.27	^***^
Sugary foods (g)	57.3^a^	41.8^b^	40.0^b^	^***^
Sweet-tasting beverages (g)	55.3	46.2	37.7	^***^

^$^BQS, BQS balanced model. ^***^p < 0.001. Same index letters (e.g., a and a) indicate that there is no significant difference between the two tertiles, and different index letters (e.g., a and b) indicate that the difference is statistically significant between the two tertiles. No indexes indicate that the difference is significant between the three tertiles (Tukey's range test).

#### 3.3.3. Sensitivity through three examples of breakfasts

**Figure 5:**
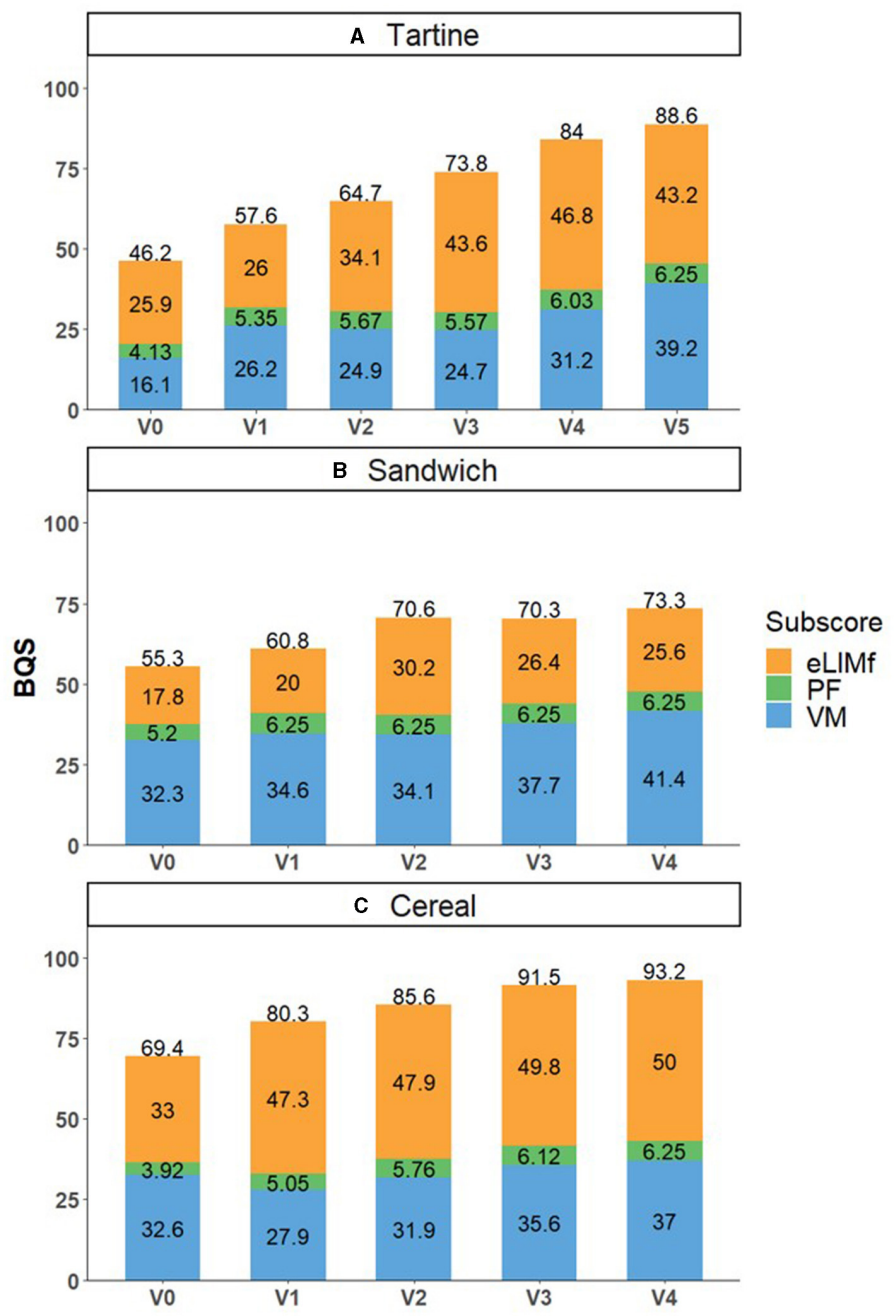
BQS and its sub-scores PF, eLIMf, and VMn for three types of breakfast: “tartine” **(A)**, “sandwich” **(B)**, and “cereal” **(C)** and their improved versions. BQS, BQS balanced model.

The sensitivity of the BQS was tested using three alternative breakfasts. Breakfast 1 (“tartine”) was a baguette with jam; Breakfast 2 (“cereal”) was ready-to-eat (RTE) cereal and milk; and Breakfast 3 (“sandwich”) was a savory sandwich. Four to five versions of each breakfast (v0, v1, v2, v3, v4, and v5) were constructed (Table 4). Breakfast nutrient content was calculated using the “Breakfast Calculator” online tool, developed by our group and available at https://ms-nutrition.com/web-app/breakfast-calculator/. The version used for the article contained the nutritional composition of foods that corresponded to the dietary intake data (INCA3) and has since been updated to reflect the most up-to-date food composition database (i.e., CIQUAL 2020). None of the eLIMf sub-scores were negative, so BQS corresponded to the stacking of the sub-scores in Figure 5.

**Table 4:** Breakfast examples.

**Breakfast type**	**Food 1**	**Food 2**	**Food 3**	**Food 4**	**Food 5**	**Food 6**	**Food 7**	**Food 8**
Tartine (v0)	French baguette (80 g)	Butter, unsalted (16 g)	Strawberry jam (30 g)	Black coffee (200 ml)				
Tartine (v1)	**Whole grain baguette** (80 g)	Butter, unsalted (16 g)	Strawberry jam (30 g)	Black coffee (200 ml)				
Tartine (v2)	Whole grain baguette (80 g)	**Dairy spread, 39–41% fat** (16 g)	Strawberry jam (30 g)	Black coffee (200 ml)				
Tartine (v3)	Whole grain baguette (80 g)	Dairy spread, 39–41% fat (16 g)	Strawberry jam (**15 g**)	Black coffee (200 ml)				
Tartine (v4)	Whole grain baguette (80 g)	Dairy spread, 39–41% fat (16 g)	Strawberry jam (15 g)	Black coffee (200 ml)	**Average fruit (100 g)**			
Tartine (v5)	Whole grain baguette (80 g)	Dairy spread, 39–41% fat (16 g)	Strawberry jam (15 g)	Black coffee (200 ml)	Average fruit (100 g)	**Plain yogurt (125 g)**		
Sandwich (v0)	French baguette (100 g)	Roasted chicken (60 g)	Butter, unsalted (16 g)	Mayonnaise (20 g)	Tomato (20 g)	Lettuce (5 g)	Black coffee (200 ml)	
Sandwich (v1)	**Whole grain baguette** (100 g)	Roasted chicken (60 g)	Butter, unsalted (16 g)	Mayonnaise (20 g)	Tomato (20 g)	Lettuce (5 g)	Black coffee (200 ml)	
Sandwich (v2)	Whole grain baguette (100 g)	Roasted chicken (60 g)	**Low-fat butter** (16 g)	Mayonnaise (20 g)	Tomato (20 g)	Lettuce (5 g)	Black coffee (200 ml)	
Sandwich (v3)	Whole grain baguette (100 g)	Roasted chicken (60 g)	Low-fat butter (16 g)	Mayonnaise (20 g)	Tomato (20 g)	Lettuce (5 g)	**White coffee** (200 ml)	
Sandwich (v4)	Whole grain baguette (100 g)	Roasted chicken (60 g)	Low-fat butter (16 g)	Mayonnaise (20 g)	Tomato (20 g)	Lettuce (5 g)	White coffee (200 ml)	**Orange (80 g)**
Cereal (v0)	Cereal, chocolate, enriched (45 g)	Semi-skimmed milk (150 ml)	Tea, no sugar (200 ml)					
Cereal (v1)	**Cereal, whole-wheat, low-sugars, fortified** (45 g)	Semi-skimmed milk (150 ml)	Tea, no sugar (200 ml)					
Cereal (v2)	Cereal, whole-wheat, low-sugars, fortified (45 g)	Semi-skimmed milk (150 ml)	Tea, no sugar (200 ml)	**Strawberry (50 g)**				
Cereal (v3)	Cereal, whole-wheat, low-sugars, fortified (45 g)	Semi-skimmed milk (150 ml)	Tea, no sugar (200 ml)	Strawberry (50 g)	**Sunflower seed (7 g)**			
Cereal (v4)	Cereal, whole-wheat, low-sugars, enriched (45 g)	Semi-skimmed milk (150 ml)	Tea, no sugar (200 ml)	Strawberry (50 g)	Sunflower seed (7 g)	**Almond (10 g)**		

Bold text indicates that the food or the amount of food is new compared to the previous version of the BF.

The balanced BQS values improved with increasing versions except between v2 and v3 in “sandwich” breakfast, where they slightly decreased. For the “tartine” and “sandwich” breakfasts, BQS increased from 46.2 and 55.3% (v0) to 57.6 and 60.8% (v1), respectively, because it enabled the fiber recommendation to be met (Supplementary Figures 1, 2). For the “tartine” breakfast, changes between v1 and v2 (64.7%) and v2 and v3 (73.8%) aimed at increasing the eLIMf sub-score, and changes between v3 and v4 (84%) and v4 and v5 (88.6%) aimed at increasing the VM sub-score. Adding an orange and plain yogurt in v4 and v5 increased calcium and vitamins C, B1, B2, and B12 while staying within the recommended energy range.

For the “sandwich” breakfast, the macronutrient profile was more favorable thanks to the replacement of butter with low-fat butter from v1 (60.8%) to v2 (70.6%), even though the mean micronutrient adequacy decreased. The addition of milk to the coffee (i.e., white coffee instead of black coffee) in v3 decreased the total score because the increases in mineral and vitamin contents brought by the milk, such as calcium and vitamin B2, were not sufficient to compensate for the increase in energy and SFA. On the other hand, adding oranges increases the score even if it adds energy to the breakfast.

For the “cereal” breakfast, the balanced BQS increased from 69.4 (v0) to 80.3% (v1) because v1 replaced chocolate cereals with low-sugar cereals, which reduced free sugars to below the threshold level. Adding fruits, almonds, and sunflower seeds in v2, v3, and v4 improved fiber, potassium, and calcium adequacy (Supplementary Figure 3). The cereal breakfast v4 was the only one to restrict eLIMf (nutrients to limit) and therefore scored the highest overall (93.2%) among the 16 breakfasts (Figure 5). The last versions of “sandwich” and “tartine” breakfasts were still too high in sodium (Supplementary Figures 1, 2).

## 4. Discussion

Nutrient profiling methods, initially developed for individual foods, can also be used to assess the nutritional value of meals. This article introduces a new way to assess breakfast quality that was specifically designed to follow a set of published recommendations for the breakfast meal from the IBRI consortium (11, 12). The BQS that was selected from among four alternatives showed the highest correlations with the NRF9.3 index and low correlations with energy density. The present BQS score is composed of three distinct sub-scores. Each sub-score had been used in previous nutrient profiling models but in different ways. The PF (protein and fiber) component has been used by nutri-score, HSR, and the NRF9.3 nutrient density index (6–8). The eLIMf sub-score was close to the negative LIM sub-score (saturated fat, added sugar, and sodium) used in NRF9.3, but with the addition of energy, a feature shared with nutri-score. The nutri-score version of eLIMf includes energy and total sugars.

The novelty here was to create a variable VMn sub-score where n varied from 0 to 14 and different permutations of nutrients were deployed. The present approach differs from that of nutri-score, which does not include vitamins or minerals but awards points for the content of fruits, vegetables, legumes, nuts, and seeds. The number of vitamins and minerals was based on the IBRI recommendations. The performance of VMn scores was tested when the number of vitamins and minerals was allowed to vary from 0 to 14. This was done to determine the minimum number of vitamins and minerals that are necessary to assess breakfast quality. It was found that less than three vitamins and minerals should be critical to derive a robust BQS. The best choice is to conserve the 14 micronutrients. However, not all food databases, especially those currently available for low- and middle-income countries, have a full set of nutrients, so our results demonstrated that using <14 micronutrients could be a viable alternative. The choice of these nutrients should be guided by relevant public health considerations and data availability. Not all nutrients are necessarily consumed in adequate amounts in the course of a single meal. When it comes to dietary inadequacies, the Dietary Guidelines for Americans identified calcium, potassium, and vitamin D as shortfall nutrients for the United States (30). Priority nutrients for low- and middle-income countries were calcium, iron, zinc, folate, iodine, vitamin A, and vitamin B12 (31). Of course, the construction of NP models depends on the availability of nutrient composition data. For that reason, it is advantageous to have flexibility in the number of score elements (32).

In the BQS construction, the calculation of the eLIMf sub-score followed a different concept compared to other nutrient profiles, which took into account unfavorable nutrients such as saturated fats, sodium, and free sugars. In BQS, the eLIMf sub-score values ranged from zero whether nutritional content is twice the maximal recommended amount to 100 whether the nutrient contents are below the limits. To penalize breakfast with a high amount of saturated fats, free sugars, or sodium, the sub-score becomes linearly negative when nutritional content exceeds twice the recommendation. Thanks to this approach, the BQS is able to discriminate between two different breakfasts with a high amount of unfavorable nutrients.

One challenge of nutrient profiling is to adequately weight the respective contributions of positive and negative components. Some existing systems appear to be mainly driven by energy density and nutrients (33, 34). In this study, we tested four alternative weighting models for the BQS. The selected algorithm, which gave equal weight to the positive (protein, fibers, and micronutrients) and negative (eLIMf) components of the BQS, showed a low correlation of the BQS with LIM, or energy density, meaning that the selected BQS would be sensitive to changes in both positive and negative components.

The sensitivity of the BQS score to small changes in the mean composition of breakfasts was illustrated with reference to three types of breakfasts. Based on BQS score distributions in French adults, 40% (close to the first tertile, which was 43.7%) appeared to be an appropriate cut-off point to identify breakfast that could be considered nutritionally adequate. Given that the present results were based on INCA3 data in adults, further work is needed to assess breakfast quality among children and teenagers.

This study had both strengths and limitations. First, the selected BQS was based on nutrient recommendations that were breakfast-specific as opposed to daily. Second, the balanced BQS was robust, showing good performance even with a limited number of micronutrients. That will be of importance in places where comprehensive nutrient composition data may not be available. In some countries, nutrient composition data are partial, and some nutrients are missing altogether. In those cases, it is useful to have a flexible and pretested BQS that can be based on the nutrients that are available. This would allow for consistent and harmonized testing of breakfast quality across multiple locations, including low- and middle-income countries. However, we did not analyze the performance of BQS, considering particular combinations of nutrients. The performance of the proposed score needs to be explored in contexts where limited nutrient content information is available. Finally, the balanced BQS was sensitive to small improvements in breakfast quality, suggesting that it may serve as a basis to educate the population on breakfast quality.

There are some limitations. First, validity testing is difficult for meal-specific indicators. The correlations were performed with another model of nutrient density, the NRF9.3 score. With single meals (as opposed to the total diet), there are no potential health outcomes. Finally, the BQS model was applied to an adult population in a single country, France. However, the BQS is easily applicable to other breakfast meals based on national dietary surveys.

## 5. Conclusion

The present study introduces a new breakfast quality score (BQS), designed to assess the nutrient adequacy of a single meal–breakfast. Similar in structure to other compensatory nutrient profiling models, the BQS introduces a novel flexible VMn sub-score based on a variable number of vitamins and minerals. The flexibility of the BQS makes it an attractive tool for evaluations of breakfast quality in settings where comprehensive nutrient composition data are not available.

## Ethics statement

The studies involving humans were approved by Comité consultatif sur le traitement de l'information en matière de recherche dans le domaine de la santé. The studies were conducted in accordance with the local legislation and institutional requirements. Written informed consent for participation was not required from the participants or the participants' legal guardians/next of kin in accordance with the national legislation and institutional requirements.

## Author contributions

GM, RP, and MM conceptualized the study. RP and MM conducted statistical analyses and wrote the first draft manuscript. AD wrote the final manuscript. GM revised the manuscript. All authors contributed to the article and approved the submitted version.

## Supplementary material

Supplementary Figure 1 Nutrient adequacies of “Tartine” breakfasts: version 0, 1, 2, 3, 4, and 5.

Supplementary Figure 2 Nutrient adequacies of “Sandwich” breakfasts: version 0, 1, 2, 3, and 4.

Supplementary Figure 3 Nutrient adequacies of “Cereal” breakfasts: version 0, 1, 2, 3, and 4.
