# Supplementary figures and images for: A three-component Breakfast Quality Score (BQS) to evaluate the nutrient density of breakfast meals
Source: Front Nutr. 2023 Sep 28;10:1213065. doi: 10.3389/fnut.2023.1213065 (PMC10569224; doi:10.3389/fnut.2023.1213065)

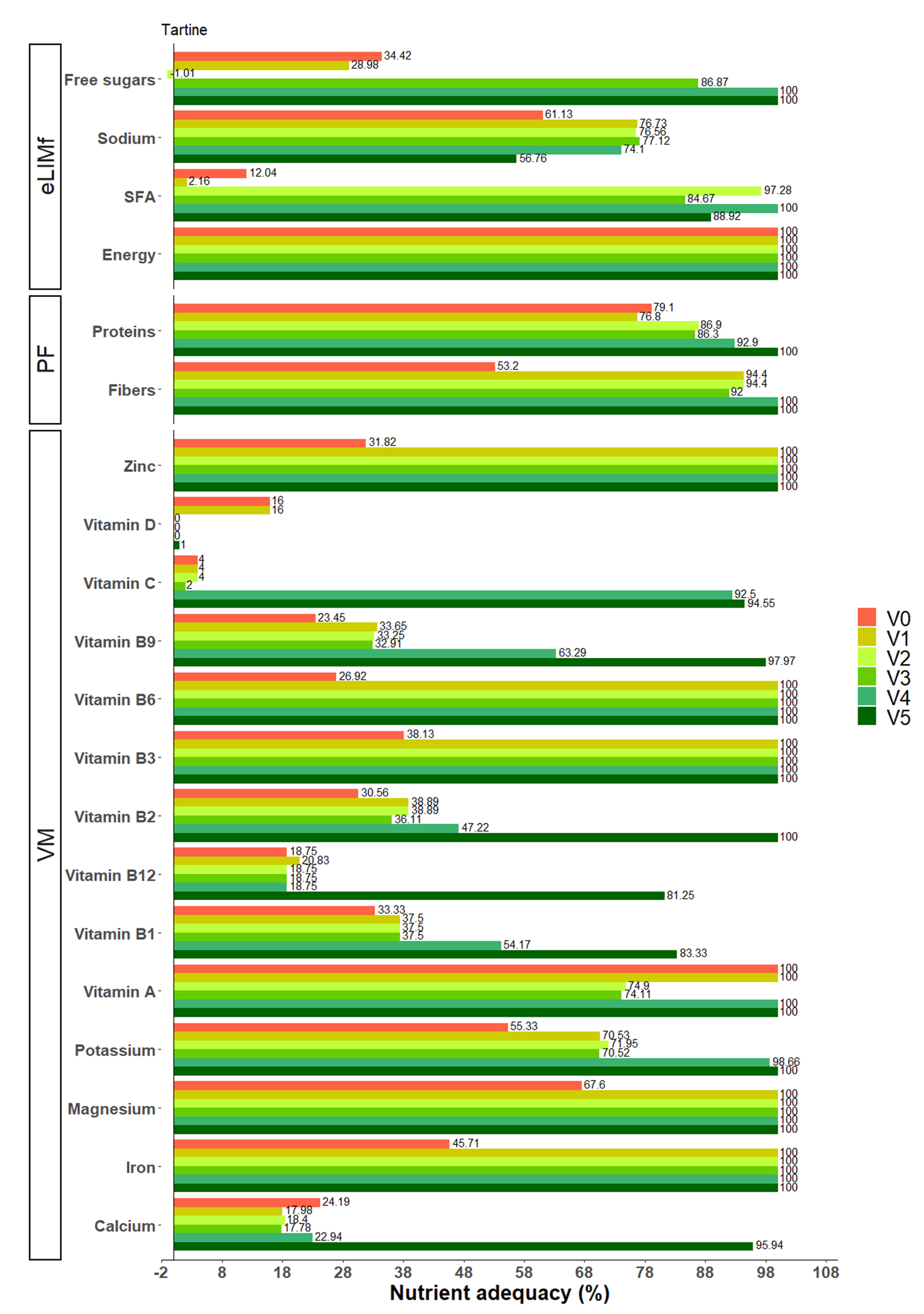

Supplement: Supplementary Figure 1 — Nutrient adequacies of “Tartine” breakfasts: version 0, 1, 2, 3, 4, and 5. [file Image_1.JPEG]

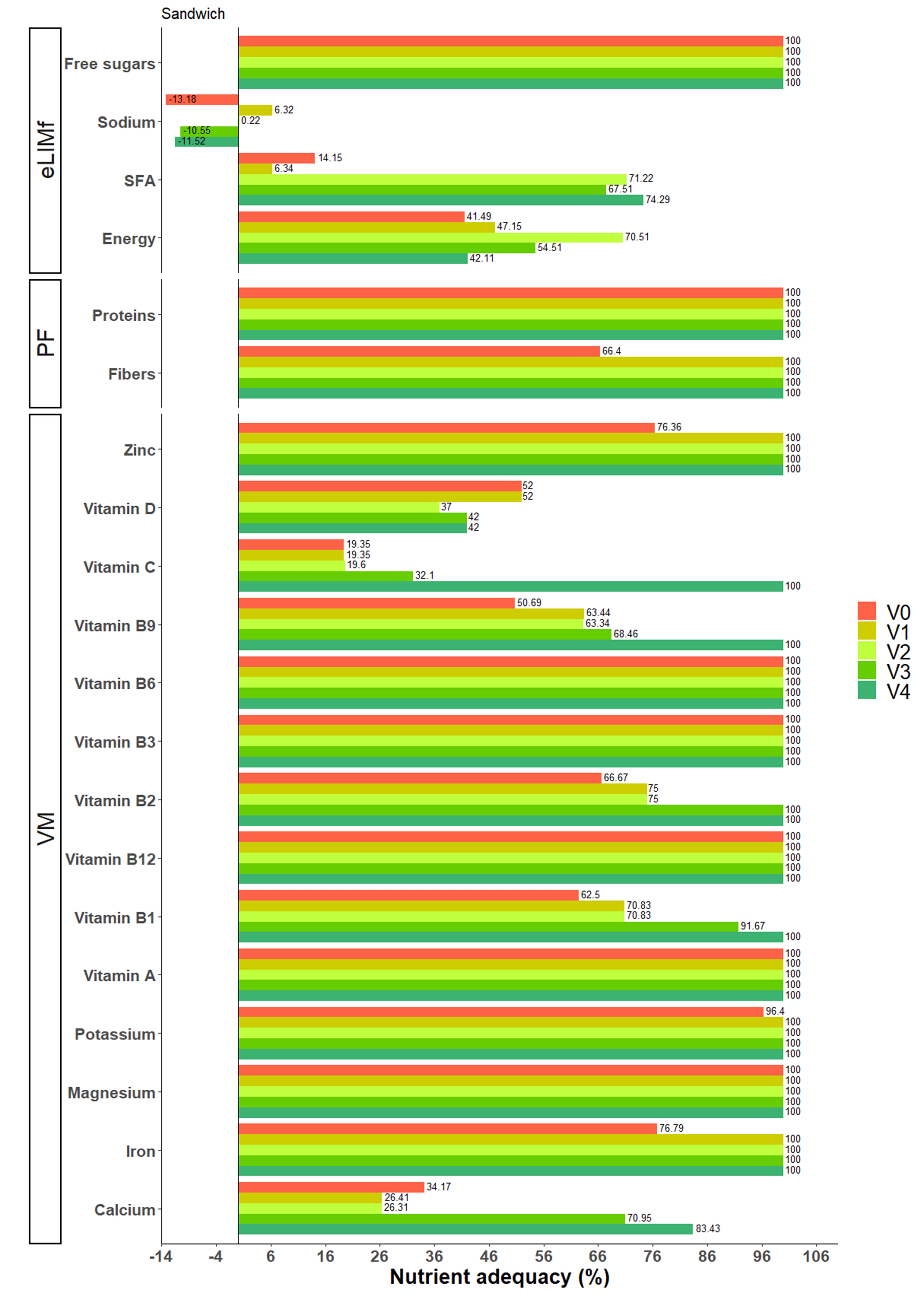

Supplement: Supplementary Figure 2 — Nutrient adequacies of “Sandwich” breakfasts: version 0, 1, 2, 3, and 4. [file Image_2.JPEG]

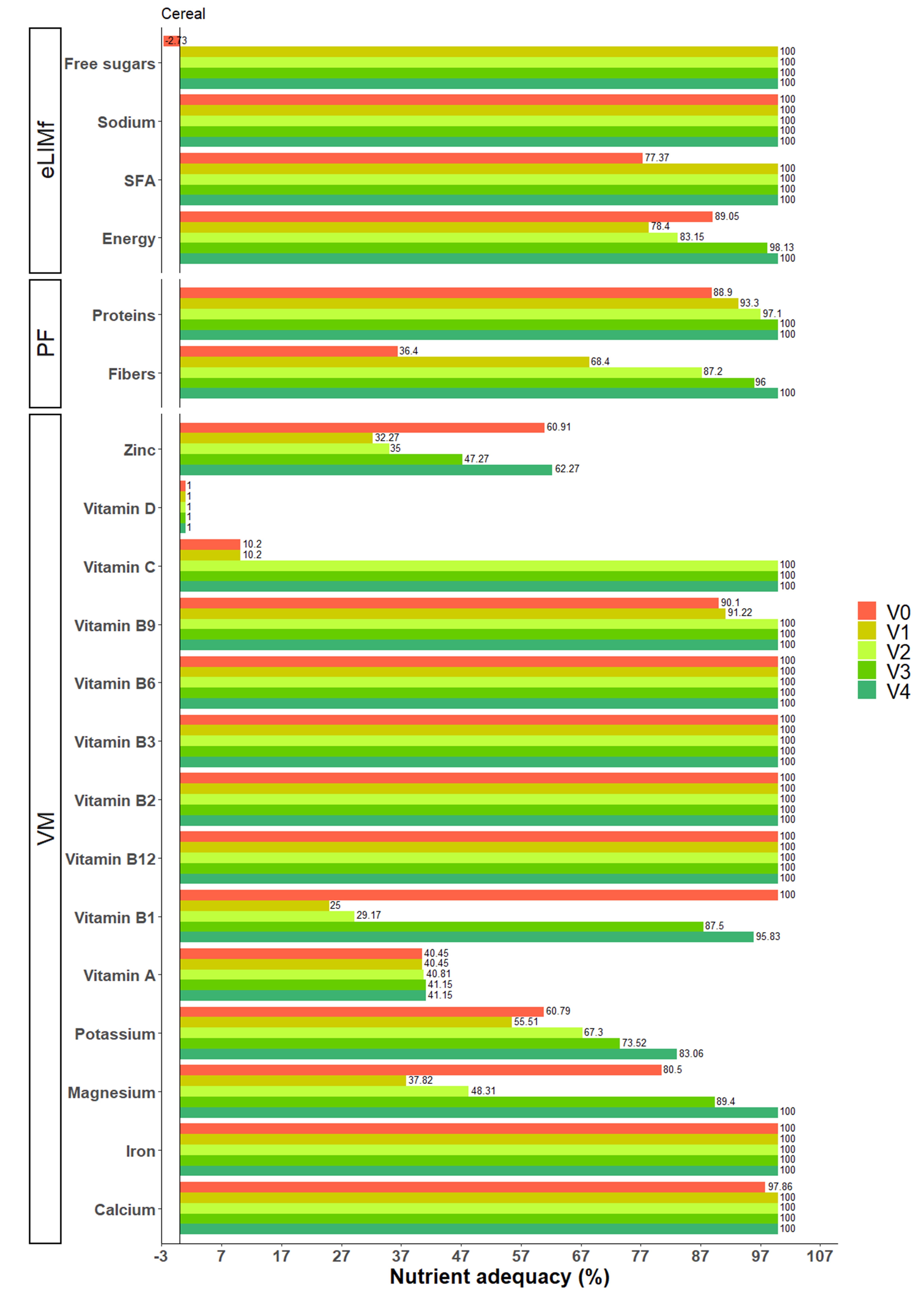

Supplement: Supplementary Figure 3 — Nutrient adequacies of “Cereal” breakfasts: version 0, 1, 2, 3, and 4. [file Image_3.JPEG]
